# Close of Volume Seven

**Published:** 1868-07

**Authors:** 


### Editorial Department.

### Close of Volume Seven.

As the present number closes the volume, we take this opportunity to thank the contributors and supporters for their generous aid, and ask a continuance of the favors we have so long and kindly received. We propose to offer a medium of communication to the members of the profession, and shall be glad to receive contributions from all who may have suggestions to make or observations to record. We have endeavored to conduct the Journal impartially in the interest of the whole profession; if it has few merits and attractions it simply shows the indifference and stupidity of the profession, and but slightly reflects upon us. The members of the profession have no right to complain of it, for it is what they make it, and if they do not regard it satisfactory, they are earnestly invited and urged to make the Buffalo Medical and Surgical Journal all they desire it to be—replete with the most valuable suggestions, the most recent discovery. We are most happy to believe that success in evidence, proof we may say, of merit, and that through all changes and embarrassments the Journal has constantly extended its scope of influence and is now stronger with its old friends and more rapidly adding new supportors, than at any previous period of its history. We are often asked for various purposes, who are the best and most reliable physicians in the various towns and villages of Western New York and adjoining parts of other States. We can turn to our subscription list, and give the desired information with most unerring certainty.

We are glad to add new names to the list of Journal supporters, and hope our friends will aid us in this object, but we do not propose any "*raid*" upon the profession for this purpose. In the city we have the names of nearly all we want, a few of the younger men who have but recently commenced practice here would be kindly received, while those who have read the Journal seven years without expense, who have never appeared upon the subscription list, or have long since *disappeared*, never giving us a manly support, are entirely welcome to *borrow* whatever more there may come to be of it; we hope its careful perusal may yet do them some good.

We anticipate for the future still greater success and usefulness, and shall spare no effort on our part to make the Journal all that its friends can desire. We shall receive the aid of the intelligent and active men of the profession, and with their assistance success is certain.

